# FunduScope: a human-centered, machine learning–based interactive tool for training junior ophthalmologists in diabetic retinopathy detection

**DOI:** 10.3389/fdata.2026.1676922

**Published:** 2026-03-13

**Authors:** Sara-Jane Bittner, Michael Barz, Daniel Sonntag

**Affiliations:** 1 German Research Center for Artificial Intelligence (DFKI), Interactive Machine Learning, Oldenburg, Germany; 2 Applied Artificial Intelligence, University of Oldenburg, Oldenburg, Germany

**Keywords:** cognitive load, design thinking framework, e-learning, human-centered design, learning tool, machine learning, usability

## Abstract

Interpreting fundus images is an essential skill for detecting eye diseases, such as diabetic retinopathy (DR), one of the leading causes of visual impairment. However, the training of junior doctors relies on experienced ophthalmologists, who often lack the time for teaching, or on printed training materials that lack variability in examples. In this work, we present FunduScope, an interactive human-centered learning tool for training junior ophthalmologists, which is based on a pre-trained ML model for classifying DR. In a qualitative pre-study, we investigated the needs of junior doctors and identified gaps in recent learning procedures. In the main mixed-methods study, we examined the experience of 10 junior doctors with the tool and its impact on cognitive load, usability, and additional factors relevant to e-learning tools. Despite technical constraints our results confirm the potential of using an ML-based learning tool in medical education, addressing the time constraints of ophthalmologists, and providing learning independence for junior doctors. However, future work could extend the learning tool by using explainable artificial intelligence (XAI) to further support the clinical decision making of learners and exceeding the scope of this proof of concept to other ophthalmic diseases.

## 1 Introduction

Junior doctors in ophthalmology learn how to interpret fundus images, i.e., images of the retina of the eye, during their medical training. Fundus images are used for the detection of DR, which is one of the leading causes of visual impairment in today's society (Teo et al., 2021). Experienced ophthalmologists teach the interpretation of fundus images, which means that junior doctors are dependent on the teaching style and methods of individual experts. However, in practice, healthcare professionals lack time for teaching and providing feedback (Glöser, 2019). Additionally, medical reasoning is complex and can result in a high cognitive load, which requires related learning materials to be well-structured (Eva, 2005). At the same time, ML algorithms in the context of DR have been shown to successfully detect pathologies such as Micro Aneurysms and classify the severity level of DR (Nichols et al., 2019; Benzamin and Chakraborty, 2018; AbdelMaksoud et al., 2020). Such models, with the ability to identify pathologies, could be used to generate training examples and provide feedback to junior doctors in training. In this work, we aim to bridge the gap between junior doctors' demand for training resources and their availability by developing FunduScope, an interactive, web-based training tool that integrates an existing ML model for detecting and locating relevant pathologies on fundus images for the detection and diagnosis of DR (Tusfiqur et al., 2022). We develop FunduScope using the human-centered Design Thinking Framework (DTF), an iterative process for addressing novel problems with an innovative approach (McLaughlin et al., 2019). With that, our work focuses on the specific teaching and learning processes of the investigated eye clinics; it aims to provide a tailored ML-based learning tool that meets the specific needs of junior doctors in our use case. A key goal when designing a learning tool is to minimize extraneous cognitive load, i.e., the load induced by the learning materials or tools themselves. The corresponding Cognitive Load Theory (CLT) describes the processing capacity that a learner possesses when solving a problem (Kalyuga, 2011), which is crucial for processing and storing knowledge. Poor usability can cause extraneous cognitive load, thereby hindering learning success. Hence, the two main aspects considered in deriving the requirements of the learning tool are cognitive load and usability. We follow the 10 usability heuristics from (Nielsen 2020) for the design and development of FunduScope to ensure high usability and low extraneous cognitive load. Additional important factors concerning e-learning include variability in practice materials, feedback, and independent practice (Davids et al., 2015). In summary, we investigate the following research questions concerning the integration of ML models in medical education, cognitive load and usability, and the additional identified e-learning factors:

- **RQ0** How does the learning tool perform in teaching the interpretation of fundus images in ophthalmology?
- **RQ1** How well does the learning tool perform in regard to cognitive load during learning the interpretation of fundus images?
- **RQ2** How well does the learning tool perform usability-wise for learning the interpretation of fundus images?
- **RQ3** How well does the learning tool perform with key components of (e-)learning for learning the interpretation of fundus images?

## 2 Related work

In this paper, we design and implement an ML-based learning tool for ophthalmologists concerning the detection of DR. Next, we introduce the disease DR, discuss its detection, and present relevant background on ML and e-learning in medicine and the two theories guiding our human-centered design process: cognitive load and usability.

### 2.1 DR disease, detection and medical training

DR is a disease of the retina caused by the patient's condition of Diabetes Mellitus. It is considered the leading cause of blindness and visual impairment in adults between 20 and 74 years (Teo et al., 2021). Regarding the increasing number of DR cases, early detection and diagnosis are becoming more important. DR can occur at 5 severity levels (0–4), which are detected and diagnosed, among other methods like the slit lamp or OCT-imaging, through an examination of a fundus image, i.e., an image of the retina (Vujosevic et al., 2020). An affected retina presents various pathologies, including Hard and Soft Exudates, as well as bleeds such as Micro Aneurysms and Hemorrhages. Regarding training, the medical field experiences a lack of medical professionals, which leads to a lack of time for teaching and feedback activities (Vaona et al., 2018). Technical tools can help bridge the gap between learning needs and the teaching methods offered. A study by (Yarmand et al. 2024) investigated the mechanisms of feedback exchange and how technical solutions can be utilized to reduce the resources required per resident in teaching for radiotherapy.

### 2.2 ML in medicine

ML, particularly computer vision techniques, is used in a wide range of medical fields. Examples include detecting relevant lesions and classifying diseases based on imaging (Nichols et al., 2019). Common problems in ML for medicine are being investigated, opening up new opportunities for the field. For example, (Kadir et al. 2023) propose the active selection of training examples for managing sparse datasets. Another work addresses the high domain dependence of models for medical imaging with base models that can be fine-tuned to the target domain (Nguyen et al., 2023). Various studies have addressed the detection of DR using ML solutions. For example, studies have been able to detect specific pathologies in fundus images, such as exudates (Benzamin and Chakraborty, 2018) or hemorrhage (Grinsven et al., 2016). These advances open up opportunities for ML-based clinical decision support (Bach et al., 2023; Carmichael, 2024). For instance, (Carmichael 2024) examined the application of human-centered artificial intelligence for decision support in ophthalmology. This aligns with proposed systems for Computer-Assisted Diagnosis (CAD) that detect DR severity and provide a visual explanation, considering explainable artificial intelligence (AbdelMaksoud et al., 2020).

In this work, we utilize a computer vision model from the literature to develop our learning tool. We apply the model introduced by (Tusfiqur et al. 2022), which implements the DRG-AI system, treating the localization of lesion areas and the DR classification as interdependent tasks. Both processes inform each other, thereby increasing the accuracy of the model. The model is trained using three datasets that provide annotations, including the severity level and the pathologies of DR: The Indian Diabetic Retinopathy Image Database (IDRiD) (Porwal et al., 2018), FGADR (Zhou et al., 2020), and EyePACS (EyePACS, 2021). The DRG-AI system is trained to predict the disease severity (in 5 stages) of diabetic retinopathy based on the corresponding clinical guidelines. It detects four pathologies first: Micro Aneurysms, Hemorrhages, Soft Exudates, and Hard Exudates. Then determines the severity based on their prominence. These four features were selected because they represent the clinically most significant and recognized indicators of DR progression, and their use aligns with prior AI-based methods, allowing for a consistent and fair comparison across studies.

### 2.3 Human-centered design in e-learning for medicine

Based on a definition of (Tirziu and Vrabie 2015), e-learning is “*the development of knowledge and skills through the use of information and communication technologies (ICTs), particularly to support interactions [...] with [...] learning activities [...].”* The use of e-learning activities in the medical field has increased in recent years (Vaona et al., 2018). However, activities in ophthalmology remain sparse.

In this work, we followed the DTF (Dam and Siang, 2021) to understand and address the needs of junior doctors when designing our e-learning tool. The DTF is an effective problem-solving framework that explores user needs and novel solutions to underexplored problems through test and iteration (McLaughlin et al., 2019). It has been applied to a wide variety of use cases, including the design of innovative medical interfaces (Goldfield et al., 2012). The framework includes five stages: (1) Emphasize: Gaining understanding of the user and the current status. (2) Define: A problem and solution statement. (3) Ideate: The goal of the Ideate stage is to diverge and create several ideas for potential solution designs. (4) Prototype: The designs are used to create prototypes that enable fast-paced testing. (5) Evaluate: The developed prototype is tested with users to gather feedback and insights.

The following section presents five key factors that emerged as relevant factors for successful e-learning in the current literature (Davids et al., 2015): Variability in Practice Material, Feedback, Independent Practice, Cognitive Load, and Usability.

#### 2.3.1 Variability in practice material

Learning with a wide variety of cases can improve the success of e-learning activities (Paas, 1992). High variability of examples facilitates the transfer of knowledge, which supports the transfer of information to different scenarios and facilitates information retrieval (Quilici and Mayer, 1996; Paas, 1992). However, the current material in the medical field remains sparse, as real-life examples need to be lavishly annotated by medical professionals to create solution sheets (see Section 2.1). That is why junior doctors in ophthalmology practice with a limited set of examples. Improving the variability of training with ML-generated examples in ophthalmology could facilitate the transfer of their knowledge to novel situations.

#### 2.3.2 Independent practice

Independent practice was named as one important factor for successful e-learning (Davids et al., 2014). Aligning with that, studying independently and at one's own pace is one of the main advantages of e-learning (Choudhury and Pattnaik, 2020). Considering the medical training in ophthalmology, independent practice is possible to some extent: Some material and practice examples can be utilized to learn independently. However, based on our qualitative pre-study most training occurs in real-time patient scenarios, with feedback provided only if time permits. This is especially affected by the limited time resources that health care professionals have (Vaona et al., 2018). Junior doctors are dependent on the specialists' teaching style and time resources. Therefore, a learning tool could improve the independent learning of junior doctors.

#### 2.3.3 Feedback

(Hattie and Timperley 2007) defined feedback as “*information provided by an agent regarding aspects of one's performance or understanding”*. In this work, we focus on task feedback as an automated learning tool that has the best insight into the factual task results (Wisniewski et al., 2020). Feedback is linked to having a positive impact on learning (Wisniewski et al., 2020; Hattie and Timperley, 2007). However, negative or uninformative feedback was highlighted to decrease self-efficacy and autonomy, which then hinders the potential positive effects (Ryan and Deci, 2000). This is why feedback should be constructive and tailored to the learner (Davids et al., 2015). Furthermore, digital advances open up new possibilities for automated feedback in e-learning, as personalized feedback can be provided without human intervention. It is linked to a range of advantages, including fast and consistent results (Alruwais et al., 2018), immediate grading, and improvement in student engagement (Marwan et al., 2020). Additionally, it enables the formation of individual learning paths based on students' prior knowledge (Ennouamani and Mahani, 2017). However, automated feedback also raises a range of concerns, such as difficulties in assessing the quality of the feedback (Kurdi et al., 2020), and the lack of implemented personalized feedback that could be helpful to students who need more support (Jensen et al., 2020). Regarding medical training, where individual feedback is often cut short in the daily process due to time constraints (see Section 2.1). Automated feedback could address the main associated challenges with feedback: Scalability and time (Henderson et al., 2019). Providing automated ways to increase the amount of feedback for junior doctors might enhance their learning outcomes.

#### 2.3.4 Cognitive load

The CLT describes the processing capacity that a learner possesses when solving a problem. It considers the limited amount of information the working memory (WM) can hold (Kalyuga, 2011). Studies indicate that through changes in the instruction of the learning material, the capacity that the capacity of the WM can be increased, which is beneficial to the learning process (Sweller et al., 1990). Furthermore, long-term memory (LTM) possesses an unlimited capacity to store learned content for a prolonged duration. The learned relations between facts can be stored as the so-called schemas, which can be more easily recalled by the learner (Sweller, 2010). An important aspect to build schemas is the variability of practice examples (Paas, 1992): Leaning on an example by (Young et al. 2014), doctors will only identify DR securely after seeing its representation in several severity levels and a combination of pathologies for each grade. In general, two types of cognitive load can be distinguished (Kalyuga, 2011). Intrinsic load refers to the load caused by the content that is learned itself (Sweller and Chandler, 1994). Extraneous load refers to the load caused by the way the task is instructed. Through these characteristics, the extraneous load can be altered more easily by modifying the task instructions. Based on the CLT and the two types of load, several effects emerged that should be considered as design implications for a learning tool: First, the Isolated Elements Effect targets the intrinsic load and suggests splitting content with a high complexity to stay within the processing capacity of the WM (Pollock et al., 2002). Furthermore, regarding the reduction of extraneous load, two effects are considered: The Redundancy Effect describes how providing information multiple times can impose a load on working memory (Kalyuga et al., 2004). Thus, only the minimal information needed should be provided by the tool. The Split-Attention Effect describes how placing related contents close to each other supports the joint processing of it (Sweller et al., 1990).

#### 2.3.5 Usability

Several studies identify usability as an important factor for e-learning (Costabile et al., 2005; Ardito et al., 2006). Usability is defined in the international standard ISO 9241-11:2018 as “*the extent to which a product can be used by specific users in a specific application context to achieve specific goals effectively, efficiently, and satisfactorily”* (Ergonomics of Human-System Interaction, 2018). Although usability has been identified as an important factor for e-learning in several studies (Freire et al., 2012; Gunesekera et al., 2019), it is still often not considered in the development and evaluation of medical training (Sandars, 2010). For example, it was observed that poor usability can limit the effectiveness of e-learning interventions (Sandars, 2010). Based on the implications of usability for e-learning design, a range of guidelines have been established, including the 10 usability heuristics by (Nielsen 2020). Relevant heuristics for developing a learning tool in the medical field are, for example: Heuristic 1, Aesthetic and Minimalist Design, highlights that only necessary information should be displayed in the interface. Adding redundant components could distract from relevant information. Furthermore, the heuristic Visibility of System Status states that users should always be aware of what is currently happening in the system.

## 3 Development of the funduscope learning tool

This paper aims to develop a learning tool for interpreting fundus images following a human-centered approach. Similar to (Sechayk et al. 2024) and (Yarmand et al. 2024), we develop a learning tool by conducting a qualitative study to derive design implications in the first step, followed by the implementation and evaluation of the tool through a user study. For that purpose, the DTF is applied as introduced in the related work (see Section 2.3). We present the steps Emphasize, Define, Ideate, and Prototype as part of this section. The Evaluation step is covered in the next section.

### 3.1 Emphasize

In the Emphasize stage, we aim to understand the current state of teaching and the junior doctor's experience when learning to interpret fundus images through a pre-study. Additionally, we aim to investigate the number of elements that doctors need to consider during a DR detection.

#### 3.1.1 Participants

The pre-study has been conducted with 7 participants. These can be further divided into two subgroups: Junior doctors and specialists in ophthalmology. Three junior doctors participated, all of whom were between 27 and 31 years old (*M* = 28.7). Two were male and one was female. They were in between their second and fourth years of training. Secondly, four specialist ophthalmologists participated who were between 31 and 48 years old (*M* = 36.8). The gender distribution was balanced with two male and two female participants. They had between six and twenty years of experience (*M* = 10.6). The participants were recruited in collaboration with the research center of the eye clinic *Anonymized Clinics*.

#### 3.1.2 Process

In the pre-study, participants signed a participant consent form. First, all participants were asked a set of questions about their demographics and experience in ophthalmology. Then, a fundus image was interpreted, and a Think-Aloud Task was applied (Cotton and Gresty, 2006), in which participants were asked to verbalize their thoughts during the interpretation. The displayed fundus image represented a case with a DR of grade 2. The results were later analyzed by a Hierarchical Task Analysis (HTA). Lastly, a Semi-Structured Interview (SSI) (Rode, 2011) was conducted with the junior doctors, which was analyzed with a reflective thematic analysis by Clark and Braun (Clarke and Braun, 2014).

#### 3.1.3 Results

The HTA indicated that interpreting fundus images represents a task of high complexity for junior doctors, as they interact with approximately 28 elements in the medical image (see Supplementary material *Emphasize Stage - Hierarchical Task Analysis*). Additionally, two pathologies were identified that the junior doctors use as anchors to navigate to other elements in the interpretation process: exudates and bleed. Further, based on the combined results of the SSI and the HTA, three main themes were derived:

1. **Junior doctors in ophthalmology might become overwhelmed by the teaching method and the high complexity of learning how to interpret fundus images**. The teaching method for junior doctors might not be ideal for their level of knowledge in medical training. The learning process is described by multiple participants as follows: “*it is always the case that the junior doctor examines the patient beforehand and [that] is then presented by the junior doctor and discussed with the ophthalmology specialist”* in the patient-doctor encounter. It is stated that “*[the interpretation of fundus images is] not really taught to you”* (P1). This lack of instruction may become challenging for junior doctors in their early training, as they often lack domain-specific knowledge. Participants wished for “*training courses that really repeat the basics from the very beginning”* (P2) and shared that “*it was expected that you can do all this already, even though you were actually in the middle of your training”* (P1). This quote highlights the gap between the actual knowledge of junior doctors at the beginning of their training and the expectations held by the training facility. From the perspective of the CLT, demanding information that the junior doctors do not have stored as schema yet can lead to an increased cognitive load (see Section 2.3.4). This weighs heavily, as junior doctors must recognize and interpret a high number of components and pathologies. Regarding the CLT, this high number of elements can overwhelm the novice. The lack of instruction can be seen, for instance, in the statement: “*There are no introductory tasks in that sense, but rather you're thrown right into the interpretation”* (P3). Aligning with this, one participant describes: “*sometimes I felt overwhelmed”* (P2).
2. **Feedback for junior doctors is restrained due to lack of time**. Junior doctors get their feedback from ophthalmology specialists as described by one participant: “*Feedback is provided by the senior physician, who may explain to you whether your own findings are correct, and in particular whether he/she has seen any additional pathological findings” (P2)*. The knowledge that is shared by specialists is perceived as highly valuable: “*when the attending describes it. This is most helpful”* (P3). However, the specialists are often restrained in their time to give feedback due to “*the shortage of time in the clinic's daily routine”* (P2) “*felt unappreciated and sometimes overwhelmed” (P2)*. This weighs especially heavily as feedback was identified as one of the most important factors for (e-)learning (see Section 2.3).
3. **The quality of teaching is dependent on the teaching ophthalmologist**. Ophthalmology specialists have a central role in the learning process: *in the end, you often need an experienced doctor who has seen certain things before and can tell you”* (P3). This weights especially strong as material was presented as insufficient for independent learning like in the statement: “*You don't really get that far with a textbook alone”* (P1) and the time for ophthalmologist specialist remain sparse: “*The scarce time in the daily routine of the clinic is also reflected in the teaching [...]”* (P1). At the same time, there is no standardized teaching procedure for their assigned junior doctors, as becomes clear from the discrepant experiences of junior doctors. One junior doctor describes a very structured experience with a “*standardized program”* (P2), while other participants describe no explanation of a complete procedure (P1) and that “*Instead, you look at it and try to see something until it's right”* (P3) drui, which describes a way less structured process.

### 3.2 Define

In the Define stage, we form a problem and solution statement based on the previous insights and derive additional requirements for the design of the learning tool.

#### 3.2.1 Problem statement

Junior doctors in ophthalmology need to learn how to interpret fundus images during their medical training to detect and diagnose diseases like DR. Learning this skill is guided by ophthalmology specialists and conducted in real patient scenarios. However, the process faces several difficulties: First, teaching directly with real patients can overwhelm inexperienced junior doctors. Based on the CLT, the lack of knowledge and the task's high complexity might exceed junior doctors' processing capacity and hinder the learning effect. Second, ophthalmologists often have limited time, which may constrain the teaching and feedback that junior doctors receive. Finally, there is no standardized teaching approach, so junior doctors depend on teaching professionals.

#### 3.2.2 Solution statement

An interactive tool that fosters independent learning could support junior doctors in ophthalmology in learning to interpret, thereby relieving the additional responsibility on ophthalmologists to teach. A wide range of training examples could be generated using the ML model by (Tusfiqur et al. 2022) with a diverse set of fundus images. With that, a wide variability of training examples is available, which supports the learning process. The ML model could classify the severity level and give data about the locations of pathologies. The junior doctor gets a training example without indications of pathologies and can draw pathologies on the image. Then, the learning tool gives feedback based on the difference between the junior doctor's input and the model's detections. This addresses several issues: It relieves teaching responsibilities from ophthalmologists, as examples and feedback are created. This further allows for the standardization of learning, so that junior doctors become less dependent on the specific teaching style of a particular doctor. Lastly, it supports junior doctors in building the necessary schemes to act confidently in interpreting fundus images beforehand and in patient-doctor encounters in the clinic.

#### 3.2.3 Requirements

We derived six major requirements based on the CLT, the 10 usability heuristics, and the e-learning factors: (1) Keep Cognitive Load to a Minimum. (2) Design a Tool with High Usability. (3) Foster the Development of Schema. (4) Foster Independence in Learning. (5) Give Tailored and Positive Feedback. (6) Give a high Variability of Examples.

### 3.3 Ideate and prototype

In the Ideate stage, the previously derived requirements are transferred to various solutions. Via Brainstorming (Wilson, 2013), we created variations for the structure, e.g., an input and feedback interface should be split structurally into two main relevant pathologies, exudates and bleeds, based on the user study in the Emphasize stage. Furthermore, we sketched the ideas from the brainstorming (Rasmussen et al., 2016). For example, the junior doctor's input and the system's feedback are displayed in one image rather than in separate ones, as displaying connected information physically close facilitates easier processing [cf. the Split-Attention Effect (Sweller et al., 1990)].

**Figure 1:**
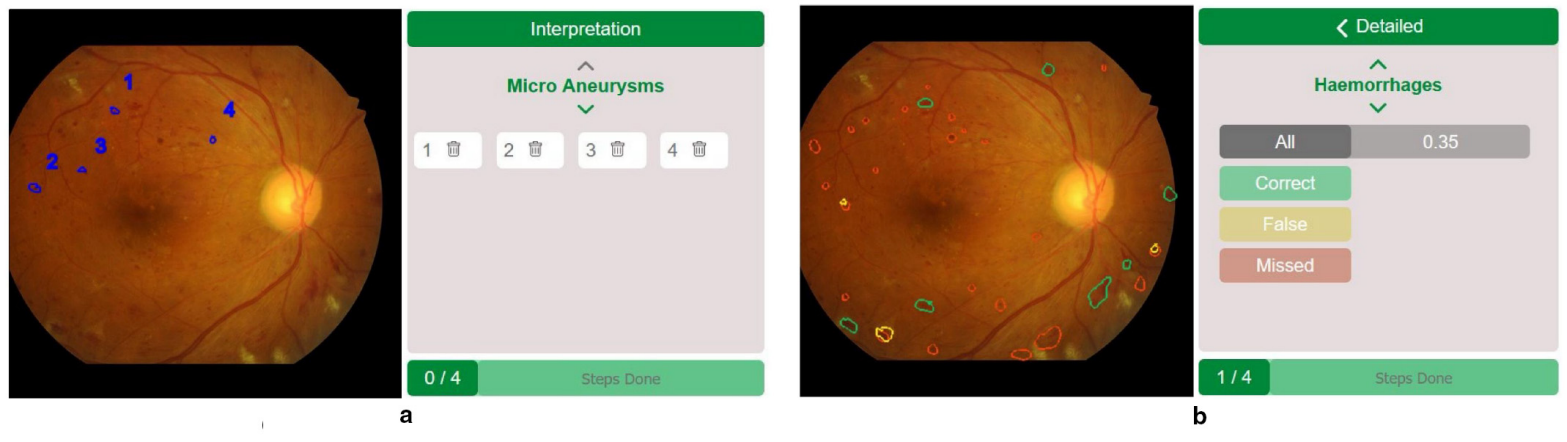
An overview of the main interaction interfaces of the learning tool. **(a)** Interpretation interface. **(b)** Feedback interface.

In this stage, we transfer the ideations to a prototype, which can then be tested iteratively. The tool was implemented as a high-fidelity prototype, as a non-technical prototype cannot sufficiently mimic the functionality of classifying pathologies. The flow of the tool can be divided into three steps: (1) Selection of the Training Examples, (2) Interpretation, and (3) Feedback. In the Selection Interface, junior doctors can choose between given examples in the Practice Area or their own examples in Extended Practice. Through the ML model that can classify pathologies in various fundus images without prior annotation, training examples with a wide range of variability can be generated, supporting the learning process (Paas, 1992). The **Interpretation interface** follows, which displays three components: The navigation bar on the left, the fundus image in the middle, and the overview area on the right. Here, the junior doctor can draw on the image to mark pathologies, each in its own color. Each pathology is displayed individually, one after another, and can be additionally navigated through the navigation bar. The structural split allows the junior doctor to focus on one aspect at a time without exceeding the processing capacity, as explained in the Isolated Elements Effect (Pollock et al., 2002). This approach might help with building the schema. The overview area provides information about the current step in the learning tool and the corresponding pathology being interpreted. Additionally, the marked lesions for the current pathology are listed here. Through the close physical display of the current pathology and the drawn lesions, the link between them becomes clearer, considering the Split-Attention Effect (Sweller et al., 1990). The Interpretation Interface is illustrated in Figure 1a.

The **Feedback Interface** displays how correctly the junior doctor interpreted the fundus image. It uses the same 3-split layout as the Interpretation Interface, which facilitates user navigation in accordance with Nielsen's fourth heuristic, Consistency and Standards (Nielsen, 2020). The overview area first displays general feedback, showing all four pathologies and the corresponding percentage of lesions that were found correctly. After clicking on a lesion, more detailed feedback becomes visible, displaying either “All,” “Correct,” “Missed,” or “False” lesions for the current pathology. The isolation of feedback types keeps the cognitive load low based on the Isolated Element Effect (Pollock et al., 2002). Additionally, the detailed information types are color-coded considering the natural understanding of users: Because of that, “correctly drawn” lesions are green, “falsely drawn” are yellow, and “missed” ones are red. This addresses requirements 1 and 2, considering high usability and keeping cognitive load low (see Section 3.2). The Feedback Interface can be seen in Figure 1b.

**Figure 2:**
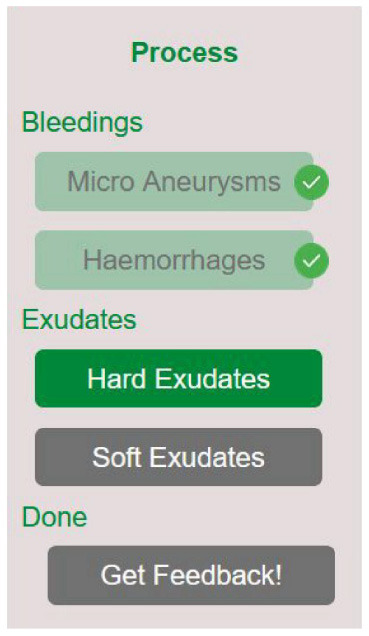
Navigation bar in the learning tool.

In the Interpretation and Feedback Interface, a navigation bar is displayed on the left (see Figure 2). It displays the four-step structure that the interpretation process follows. It leads through two types of pathologies identified by the pre-study in the Emphasize stage (Section 3.1): Bleeds and Exudates, which are then further divided. This divides the process into smaller units. Based on the Isolated Elements Effect, this helps to process and split the cognitive load induced by a task that is otherwise high in complexity (see Section 2.3.4). The navigation bar always displays the step that the junior doctor is currently in, as well as which steps have already been completed and which are still to come. This aligns with the first heuristic by (Nielsen 2020), Visibility of System Status.

#### 3.3.1 Technical implementation

**Figure 3:**
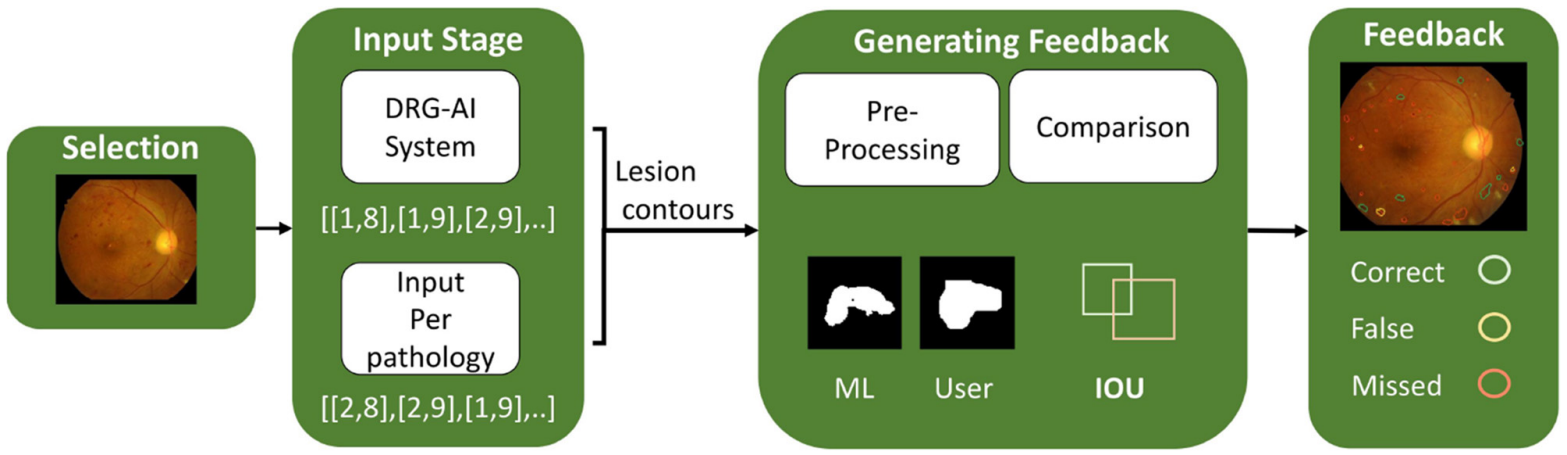
Overview of the technical architecture of the learning tool.

The FunduScope learning tool applies the DRG-AI system introduced in Section 2.2 to automatically detect pathologies (Tusfiqur et al., 2022). The model has previously been applied to CAD for DR. It supports the localization of four pathologies: Microaneurysms, Hemorrhage, as well as Soft and Hard Exudates. This lesion identification and classification ability was used to extract the contours of individual lesions for each pathology in the pixel space of an input image. The contours of pathologies marked by junior doctors via the Interpretation Interface are also saved as coordinates in the pixel space of the image. All contours, i.e., those detected by the DRG-AI system and those marked by junior doctors, are saved as images. These are processed to retrieve the pixel coordinates of the complete lesions, not just their outlines: the shapes are drawn on a plane, then the contours are dilated, and a closing morphology is applied to ensure that the shapes are fully closed. In the next step, we iterate over the lesions detected by the ML model for each of the four pathologies and compare them to the manually marked lesions. An Intersection Over Union (IOU) score is calculated for each detection lesion in comparison to the marked lesions. The higher the IOU score is, the better the lesion-pair fit. After calculating the IOU score for the detected lesion and all marked lesions of the corresponding pathology, the marked lesion with the highest IOU score is assigned as a correctly marked lesion and deleted from the list of lesions by the junior doctor. We consider a marked lesion with an IOU score of 0.5 or higher for one of the detected lesions as correct. We store these lesions as “correct.” Manually marked lesions without a corresponding ML-detected lesion are stored as “falsely input,” and the detected lesions for which we did not find a corresponding manual input are stored as “missed.” An overview of the technical model can be found in Figure 3.

## 4 Evaluation

**Figure 4:**
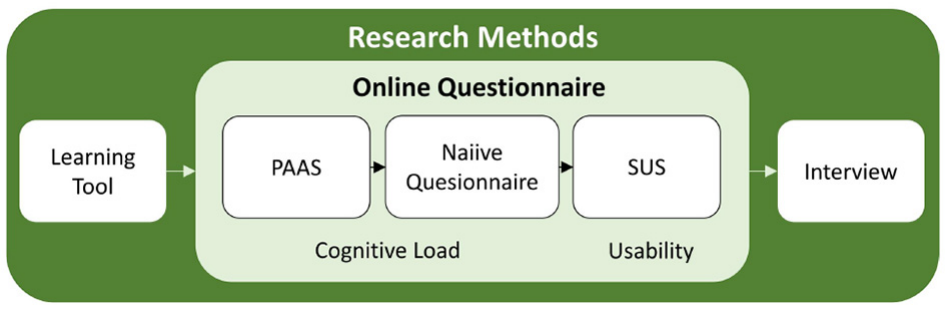
Study plan of the evaluation study.

A user study is conducted to investigate the usability and cognitive load, as well as the experiences of the junior doctors when using the learning tool. Aligning with common practice for qualitative case studies, 10 participants were recruited to conduct the study (Caine, 2016). That way, pain points and recommendations for further design can be derived. Further, quantitative results were not derived, due to the characteristics of the study as a qualitative case study, aligning with the DTF (see Section 2.3) to inform the next stage of development iteratively. An overview of the study plan can be derived in Figure 4. Ten junior doctors in ophthalmology participated in the study, who were between 27 and 35 years old (*M* = 30). They were recruited in collaboration with five national eye clinics. In regards to their medical training they were in between their first and fourth year of training with a mean of 2.6.

### 4.1 Procedure

Initially, participants sign an informed consent form. Then, the process consists of three parts: First, the participants execute two example tasks in a randomized order, annotating a fundus image with the ML-based learning tool. Then, they complete an online questionnaire, which includes two measures for cognitive load: the Paas scale and the Naiive questionnaire, as well as the System Usability Scale (SUS) to measure usability. Lastly, an SSI is conducted to evaluate user experience and e-learning factors. The study was closed with a debriefing.

### 4.2 Methods and measures

This study included measures to observe participants' cognitive load and subjective usability when interacting with the learning tool. We used two subjective rating scales to measure cognitive load. The Paas scale by (Paas 1992) consists of one item phrased: “*In solving or studying the problem I invested [...] mental effort”*. It is measured on a 9-point Likert scale. The scale gives a general overview of the cognitive load and does not distinguish between intrinsic and extraneous load. Additionally, the German version of the Naiive questionnaire by (Klepsch et al. 2017) is conducted. It distinguishes between types of cognitive load through sub-scales: Intrinsic load is queried by two items, with one example being “*This task was very complex”*, while the scale for extraneous load has three items, with one example being “*During this task, it was exhausting to find the right information”*. Both sub-scales are measured by a 7-point Likert scale. The German adaptation of the SUS was used to assess the subjective usability of the learning tool (Brooke, 1996; Rummel, 2016). The SUS contains a total of 10 items, which are measured by a 5-point Likert scale. An example item is: “*I found the system unnecessarily complex”*. The score ranges from 0 to 100, with higher values representing better usability. Furthermore, we conducted an SSI to gain insights into the experience of junior ophthalmologists using the learning tool. To analyze the SSI, a reflexive thematic analysis was applied (Clarke and Braun, 2014).

### 4.3 Results

This section reports the descriptive statistics for cognitive load and usability descriptive statistics for cognitive load and usability. An overview of these results can be taken from Table 1. Additionally, we introduce three themes from the SSI.

**Table 1:** Descriptive values of the Paas scale by (Paas 1992) and the Naiive questionnaire by (Klepsch et al. 2017) for cognitive load and the system usability scale by (Brooke 1996) for Usability.

**Descriptive values**	**Paas scale**	**Naiive questionnaire**		**SUS**
		**Intrinsic load**	**Extraneous load**	
N	10	10	10	10
Mean	3.80	1.55	1.87	76.0
SD	1.32	0.550	0.549	6.79
Min	1.00	1.00	1.00	62.5
Max	6.00	2.50	2.67	85.0

#### 4.3.1 Cognitive load

The following paragraph covers the descriptive statistics of the Paas scale and the Naiive questionnaire. An overview of these results can be taken from Table 1. For the Paas scale, the mental effort is rated rather low to medium during the use of the ML-based learning tool with a score of *M* = 3.8 (*SD*=1.32) on a 9-point Likert scale. For the Naiive questionnaire by (Klepsch et al. 2017), the sub-scale for intrinsic load reports a score of *M* = 1.55 (*Std* = 0.55), while the sub-scale for extraneous load receives a score of *M* = 1.87 (*Std* = 0.55). As 1 represents the minimum and 7 the maximum, the values for both intrinsic (*1.55*) and extraneous load (*1.87*) are on the lower end of the scale, indicating that participants are not experiencing significant mental effort.

#### 4.3.2 Usability

The *SUS* score is *M* = 76 (*Std* = 6.79, *min* = 62.50, *max* = 85.00). Based on (Bangor et al. 2009), a score between 73 and 84 represents “good” usability. An overview of these results can be taken from Table 1. Thus, the ML-based learning tool receives an above-average, good score.

#### 4.3.3 SSI

In total, 5 themes were derived from the reflexive thematic analysis of the SSI. The participant IDs do not correspond to the IDs in the pre-study.

**1. The design facilitates the use of the tool**. The junior doctors found the design to be intuitive and visually clear. The understandable design was highlighted by several participants stating that the tool is “*Very clearly and simply structured”* (P8). One main aspect was the minimalist design, which was emphasized to “*reduce the complexity”* because “*you don't have much choice [...]”* (P5). Concerning cognitive load, participants experienced the task as “*not very demanding”* (P7), which aligns with the clear design. Additionally, the use of “*simple”* (P2, P4, P9, P6) color-coding was highlighted to aid understanding and described to provide clear indications, similar to a “*traffic light”* (P1, P2, P4, P7, P9, P10).

**2. The structure of the tool supports user guidance**. The structural split into four pathologies supports the navigation of junior doctors in the tool. The intuitive navigation was highlighted by doctors stating: “*I can get started quickly, [without] instructions”* (P5) and that the flow of the tool “*was already very clear [...]* (P1). Other helpful aspects are the display of current, past and future steps in the navigation bar “*it was nice that you could see what you had already done”* (P7) and the minimalist design that highlighted relevant options “*The software only has a few options”* (P10).

**3. Technical factors limit the learning experience**. Two main technical limitations were identified during the tool's use: First, it was noted that the algorithmic solution exhibits inaccuracies in detecting certain pathologies. This led junior doctors to describe the tool as useful only “*if the recognition were better”* (P6). The second technical limitation concerns the misalignment of the comparison algorithm, which determines whether a pathology was correctly or incorrectly input by the junior doctor. One junior doctor described that “*my circles were not always made appropriately enough”* (P9), which highlighted that the algorithm was counting lesion borders more precisely than the user would input them.

**4. The type and structure of the feedback supports the learning process**. The feedback supports the junior doctors regarding two main aspects: Structure and characteristics. First, regarding the structure, the general split in pathologies supports the understanding. Aligning with this junior doctor states that “*that was well divided into categories and not all on top of each other”* (P3). Furthermore, the division of feedback information into four categories was found to be helpful. A junior doctor expressed “*So in principle four things, red, yellow, green, very clearly structured”* (P4). Through the choice of the displayed information, the junior doctor could hide clutter and focus on the relevant information for their individual learning process. Secondly, the direct timing of the feedback was pointed out: “*the software simply allows me to actually get feedback for each case again”* (P9). This was mostly argued with the lack of time that is available in clinics for feedback: “*There is not explained that much in everyday clinical practice”* (P6). Overall, the feedback on the learning tool supports the learning process. For this, it guides the doctor structurally through the process and provides them with the opportunity to focus on specific situations. Further, it offers direct feedback that is not influenced by the time constraints of the clinics.

**5. The learning tool fosters the independent learning process**. The characteristics of the learning tool support the independent learning of interpreting fundus images. Several junior doctors pointed out that seeing novel examples of the learning tool can support the learning process: “*If I've never seen it before, then I simply learn better with [the tool]”* (P1) and “*So I don't have to think up what that could be myself”* (P5). This highlights the importance of clearly displaying separated pathologies for junior doctors. Furthermore, it was emphasized that the learning tool facilitates the learning process by providing flexibility in the timing of learning. It is possible to “*take your time with a fundus”* (P10) and one participant describes: “*Yes, I think it's good, Because you can simply do it again in peace”* (P1). Aligning with that one junior doctor shared “*you don't have to invest time in searching for an answer”* (P7). Several junior doctors share that with the saved time and learning they “*could make a diagnosis earlier”* (P2) which could benefit the clinical process. Adding to that the “*self-explanatory”* (P3, P4, P5, P6, P8, P9, P10) design of the tool enables them to “*just get started”* (P8) with the learning process which increases their independence in learning. Overall, the learning tool supports the independent learning process by providing the opportunity to become familiar with concepts at the junior doctor's own pace and needs, without requiring guidance from a specialist.

## 5 Discussion

This work followed the human-centered DTF to develop a learning tool for junior doctors in ophthalmology. They must learn how to interpret fundus images during their medical training to detect and diagnose diseases such as DR, which represents the leading cause of visual impairment (Teo et al., 2021). However, the interpretation is taught by ophthalmologists, who lack time to teach and give feedback (Glöser, 2019). An ML-based learning tool is proposed to close the gap between the required teaching and available resources. The applied ML model generates training examples in a time and cost-effective manner. To elicit the human learning process, design implications for cognitive load and usability were considered for the development.

### 5.1 General RQ: How does the learning tool perform in teaching the interpretation of fundus images in ophthalmology?

In general, the key findings indicate that the learning tool performs sufficiently in teaching junior doctors in ophthalmology to interpret fundus images. The general impression of the learning tool was positive, and the junior doctors liked the understandable design and navigation. Aligning with that, the tool performed well in both scales regarding cognitive load, with low to medium values. Further, the SUS indicated a generally positive experience with the tool, reflected in a good usability score. Additionally, the ML model generates a wide variety of examples, which supports the learning process and facilitates the transfer of information, thereby promoting independent learning. However, the results indicate that the technical solution has limitations that might hinder the learning process. Certain pathologies are not detected accurately enough, leading to decreased trust in the software and limiting the learning experience. The successful building of a schema is limited due to technical constraints. This is especially crucial as the tool is applied in a medical context where inaccuracies can lead to wrong detection and diagnoses.

### 5.2 RQ1: How does the learning tool perform regarding cognitive load?

The results indicate a **suitable level of cognitive load** when using the tool. Both measures of the online questionnaire also indicate this: First, the Paas scale by (Paas 1992) shows a low to medium general mental effort, while the Naiive questionnaire by (Klepsch et al. 2017) presents values for both the extraneous and the intrinsic load that indicate lower load. In line with the results of the questionnaire, the interview themes support the outcome: Starting with theme 1, **the Design Facilitates the Use of the Learning Tool**, which indicates that the eighth usability principle (see Section 2.3.5) and the Redundancy Effect (see Section 2.3.4). This includes that if unnecessary information is added to the learning tool, it must also be processed. As the design focuses on the most important components, junior doctors can follow smoothly. Further, it is pointed out in theme 2 **The Structure of the Learning Tool Supports the User Guidance**. Here, the structural split into four pathologies was highlighted as clear and helpful for navigation. This result aligns with the Isolated Elements Effect (see Section 2.3.4). This effect implies that splitting a complex task into several isolated steps can also split the total intrinsic cognitive load. For the interpretation of fundus images, the complexity was estimated to be high based on the observation in Emphasize (see section 3.1). This is because the process of medical reasoning includes multiple elements of the retina and pathologies on the fundus image that need to be considered (Eva, 2005). Therefore, splitting the pathologies splits the processing capacity needed at each step, reducing the intrinsic cognitive load. However, theme 3 **Technical Factors Limit the Learning Experience** indicates sources for increased cognitive load in the Feedback Interface. First, the model sometimes classifies pathologies incorrectly, and second, the comparison algorithm used to evaluate the junior doctors' input against the algorithmic solutions sometimes provides inaccurate feedback. The described increase in cognitive load by junior doctors aligns with the theory's assumptions, as the inconsistencies in feedback are additional elements that need to be processed. When feedback is given based on false calculations, the mental capacities are used to distinguish between system errors and real errors. With that, the tool does not sufficiently fulfill requirement 3 “*Foster the Development of Schema”* as incorrect knowledge would be taught in these cases.

### 5.3 RQ2: How does the learning tool perform in usability for learning the interpretation of fundus images?

The results indicate a good level of usability and stress, suggesting that the structure, task, and understanding were mostly clear during use. A good usability is also confirmed by a mean SUS score of 76 points. In line with the results of the questionnaire, the interview themes support this outcome as well: Starting with theme 1 **The Design facilitates the Use of the Learning Tool**. Similar to the previous question, it indicates that the design was experienced as understandable through the minimalist design. The results build on the eighth usability heuristic (see Section 2.3.5), which suggests that when only necessary elements are displayed, they are highlighted and can be processed more easily. Additionally, it aligns with the sixth usability heuristic, Recognition rather than Recall, which states that visibly displaying all available options can reduce the difficulty of using an interface, as the user does not need to remember all options. Further, similar to the previous question, theme 2 **The Structure of the Learning Tool supports the User Guidance** aligns with the presented eighth and sixth usability heuristic: Junior doctors experience the navigation intuitively because of the learning tool's clearly structured and minimalist design. One major aspect that was pointed out is the **navigation bar**. The junior doctors expressed that due to the structure and colored elements, the flow of the learning tool was clear. This statement aligns with several usability heuristics by Nielsen (see Section 2.3.5): First, the navigation bar is placed on the left side of the learning tool and showcases the process's structural split into four pathologies. The tool adheres to navigational standards, and users are accustomed to following a flow from top to bottom, in accordance with the fourth usability heuristic. Further, the junior doctors expressed that the color coding of the navigation bar was aiding their use. Here, the navigation bar displays the current, past, and future steps differently. The potential increase in usability because of the visibility of the system status aligns with the first usability heuristic. Lastly, the interview indicated that going a step back with the navigation bar and deleting incorrectly input lesions supported the junior doctors in using the learning tool. This aligns with the third and ninth usability heuristics: User Control and Freedom and Recover from Errors. Giving the junior doctors the option to change the current pathology, go back, and re-do steps prevents them from feeling stuck in the learning tool. However, similar to the first research question, theme 3 **Technical Factors limit the Learning Experience** indicates sources for a decrease in usability in the Feedback Interface. In some rare instanced the the correctly drawn pathologies by the doctors were incorrectly classified as “Missed.” These errors in the learning tool's calculation were then included in the junior doctors feedback, which might have negatively impacted the tool's usability. It does not sufficiently fulfill requirement 3 “*Foster the Development of Schema”*, as potentially incorrect knowledge might be conveyed.

### 5.4 RQ3: How well does the learning tool perform with key components of (e-)learning for learning the interpretation of fundus images?

The results of the study indicate that, generally, the requirements regarding important e-learning factors were met, considering the aspects of feedback, independent learning, and variability of examples (see Section 2.3). First, regarding feedback, the results indicate that the split of information and color design of the feedback in the learning tool supports the learning process. This is primarily based on the results of theme 4, **The Type and Structure of the Feedback Supports the Learning Process**. Junior doctors expressed that the structural split into four pathologies supported their processing of the feedback. This aligns with the isolated elements effect as the content can be processed step by step (see Section 2.3.4). Moreover, the possibility of blending feedback out and focusing on selected information was highlighted. Aligning with the redundancy effect, hiding unnecessary information can help junior doctors focus on relevant input, leading to a lower level of extraneous cognitive load. Furthermore, the results suggest that the learning tool facilitates the independent learning of junior doctors, aligning with theme 5, **The Learning Tool fosters the Independent Learning Process**. For one, junior doctors stated that the learning tool supported them in learning to detect new pathologies and practice the concept several times to acquire knowledge. With that, it aligns with requirement 3 “*foster the development of schema”*. Due to repetition, knowledge can be stored in new schemas, which can then be accessed in clinical practice. Further, junior doctors emphasized that the learning tool is self-explanatory due to its minimalist design, which aligns with usability heuristic eight (see Section 2.3.5). Junior doctors can understand and navigate the learning tool more easily and navigate it independently. Lastly, another aspect raised by the junior doctors for independent learning is that the learning tool can be used at one's own pace and time due to the immediate feedback.

Additional attention needs to be drawn to the decrease in pressure when learning with the tool. From the emphasize stage, we know that junior doctors currently learn within in-patient scenarios without having the required information yet (see Section 3.2), which leads to increased cognitive load and stress. While stress can have a negative impact on performance and decision-making processes in health professionals' education (LeBlanc, 2009), other studies suggest that stress can be beneficial for the memory process in learning (LePine et al., 2004). As the impact of stress on e-learning was not further investigated in this work, follow-up studies should consider stress and its implications for an e-learning tool. Lastly, the results indicate that the training tool offers a wide variety of training examples. As indicated in Section 2.1, the learning tool can generate new examples and therefore increases variability. With that, it addresses the challenge of creating solution sheets manually by teaching doctors. Additionally, junior doctors can upload their own training examples.

Overall, the important e-learning factors derived in Section 2.3 are met by the learning tool. First, the feedback supports the junior doctors due to its structural design and the possibility of hiding information. The learning tool supports independent learning by providing immediate feedback, displaying high-variability training examples, and being designed to be self-explanatory, as well as to tailor the learning to one's own time schedule. However, similar to the previous research questions the interview indicated that technical limitations constrain the quality of the feedback. This is especially crucial as this is a medical application, and therefore, inaccurate teaching can lead to wrong detection and diagnoses. Therefore, the full range of performance can only be assessed after the technical limitations have been addressed.

### 5.5 Implications

Medical tasks are often complex because many variables must be considered (Eva, 2005). Furthermore, poor usability, which may increase extraneous cognitive load, has been shown to hinder success in e-learning. Consequently, learning tools should strive to minimize cognitive load while maintaining high usability. Additionally, high variability of examples supports the learning process. Based on the design process and evaluation, we derived the following implications:

1. A learning tool for a medical context should **split feedback to not overwhelm junior doctors**. The smaller portions can be processed individually, adhering to the split-attention effect as outlined in cognitive load theory (cf. Split-Attention-Effect in Section 2.3.4). Additionally, the learning tool should allow for **blending out aspects of information**. Providing the option to focus on a specific type of feedback at a time might enhance understanding, as redundant information does not need to be processed, adhering to the redundancy effect based on cognitive load theory.
2. A **minimalist design might increase usability** and **highlight important elements** of the design. **With fewer elements, the extraneous load is decreased**, which frees the processing capacities of junior doctors. Information added to the learning tool should be checked for its importance and deleted if it appears redundant (cf. Redundancy Effect in Section 2.3.4). This aligns with the eighth usability heuristics of Nielsen, i.e., Aesthetic and Minimalist Design, as well. That way, learners can focus on processing the relevant information
3. A learning tool should include a **clear navigation bar that is structured in a familiar way, visualizes the current status, and allows changing between steps of the learning process**. That way, medical learners can tailor the process to their needs and redo steps if necessary. This adheres to three of the usability heuristics by (Nielsen 2020): User Control and Freedom, Visibility of System Status, and Consistency and Standards.
4. A digital learning tool should be **self-explanatory to enable independent learning**. One main problem derived from the emphasize stage is that junior doctors are dependent on experienced doctors teaching them. The digital learning tool can provide a solution to decrease this independence by offering an additional source of teaching. A self-explanatory design can be achieved by following the usability heuristics (see Section 2.3.5). For example, the design could be kept minimalist, and the system's process can be aligned with the real-world experience of doctors.
5. An ML-based learning tool can facilitate training with a diverse set of training examples without requiring extensive time from healthcare professionals to annotate or review them. ML-based tools should be considered in the development of learning tools, while also acknowledging the negative impact of technical limitations on the learning process.

### 5.6 Limitations and future work

The results of this work should be considered in the light of some limitations. Firstly, technical limitations regarding the classification of pathologies and the evaluation of the junior doctors' input could have impacted the cognitive load and usability during the use of the learning tool. Therefore, a follow-up study should reevaluate a version of the learning tool that addresses the technical limitations. Secondly, the current version does not display the model's accuracy and does not inform the learner about potential bias in the model. In the medical context, it is crucial that doctors can accurately estimate the accuracy of the ML model to ensure safe clinical decision making. A future version of the tool should inform junior doctors about potential biases in ML systems and provide specific information about the performance of the applied ML model to avoid biased diagnoses and develop an appropriate level of trust toward the system. That way, the requirements for explainable artificial intelligence, as outlined in the General Data Protection Regulation (GDPR) of the European Parliament, are met (Sartor, 2020). Thirdly, to further support the building of schema as proposed in requirement 3, the tool could be enhanced by explainable methods. For example, XAI can be used to enhance clinical decision making by providing additional information through linking relevant literature (Yang et al., 2023) or increasing the trust and acceptability of tools (Antoniadi et al., 2021). Future work could provide junior doctors with explanations based on cognitive load and level of expertise to support the learning process (Maehigashi et al., 2024; Corti et al., 2024). Lastly, while the current model focuses exclusively on these DR-related biomarkers, it can be extended to other ophthalmic diseases such as glaucoma or age-related macular degeneration (AMD) by integrating additional disease-specific biomarkers, adopting approaches like Transfer Learning. For example, (Pascal et al. 2022) built a multi-task deep learning network that simultaneously learns segmentation and classification for glaucoma from fundus images. Similarly, joint optic disc/optic cup segmentation networks (e.g., Fu et al., 2018) illustrate how modular, multi-output lesion-level models can support downstream disease classification. Furthermore, hybrid multi-disease prediction systems (e.g., Zedadra et al., 2025) demonstrate that a unified model handling multiple ophthalmic conditions is feasible.

## 6 Conclusion

We adopted a human-centered approach to develop an ML-based learning tool for interpreting fundus images, designed for junior doctors in ophthalmology, to bridge the gap between the required knowledge in medical training and the limited time available for teaching by ophthalmology specialists. For the development, an ML model was used that can detect pathologies in fundus images. Furthermore, the DTF was followed to center the development around the needs and experiences of junior doctors. To determine the potential of an ML-based learning tool, we first investigated the current status of learning in a pre-study. We considered cognitive load because doctors need to process a lot of elements simultaneously, which can impede the learning process. For this, effects such as the Isolated-Elements and Redundancy Effect were considered to split tasks and display only necessary information. Additionally, poor usability can increase the load, thereby hindering the learning process. That is why we followed the 10 usability heuristics by (Nielsen 2020) during the design process. Further, important factors of e-learning, such as feedback, independent learning, and variability in learning examples, were considered. Finally, the ML-based learning tool was evaluated in a mixed-methods user study to identify pain points and derive recommendations for the future development of medical learning tools. It can be concluded that the developed ML-based learning tool meets the general requirements set in the research questions: First, the tool successfully kept cognitive load low by dividing tasks and simplifying information. Further, usability was enhanced through minimalist design and clear system visibility. Additionally, the e-learning factors were addressed with positive results regarding the structure and type of feedback, as well as the independence that the learning tool offers for junior doctors. Lastly, applying the ML model enabled the generation of a wide range of variable training examples, which are important for the learning process. However, limitations arose in the accuracy of classifying pathologies and the quality of feedback. This could compromise the reliability of the learning tool in a medical context. Despite these technical constraints, the study suggests that an ML-based learning tool is feasible for medical education, addressing time constraints and providing valuable learning independence for junior doctors. Learning tools, especially in medical education, should therefore, (1) Split the Complexity by Dividing Tasks, (2) Apply a Minimalist Design to Highlight Important Elements, and (3) Integrate a Clear Navigation Bar. Future studies could address the detected technical limitations and, based on that, re-evaluate the tool's impact on cognitive load as well as usability.

## Data Availability

The original contributions presented in the study are included in the article/Supplementary material, further inquiries can be directed to the corresponding author.
