# Supplementary material for: FunduScope: a human-centered, machine learning–based interactive tool for training junior ophthalmologists in diabetic retinopathy detection
Source: Front Big Data. 2026 Mar 13;9:1676922. doi: 10.3389/fdata.2026.1676922 (PMC13021406; doi:10.3389/fdata.2026.1676922)
Supplement: Supplementary file 1 [file Data_Sheet_1.pdf]

Supplementary Material

1 EMPHASIZE STAGE - HIERARCHICAL TASK ANALYSIS

The *hierarchical task analysis* (HTA) was applied in the emphasize stage to understand how junior doctors interpret fundus images and what relevant elements can be identified during the process. In general HTA is a top-down approach in which the task is represented as a primary goal and is then further divided into sub-goals with corresponding activities. For this work, the data is collected via the transcripts of a concurrent think aloud protocol.

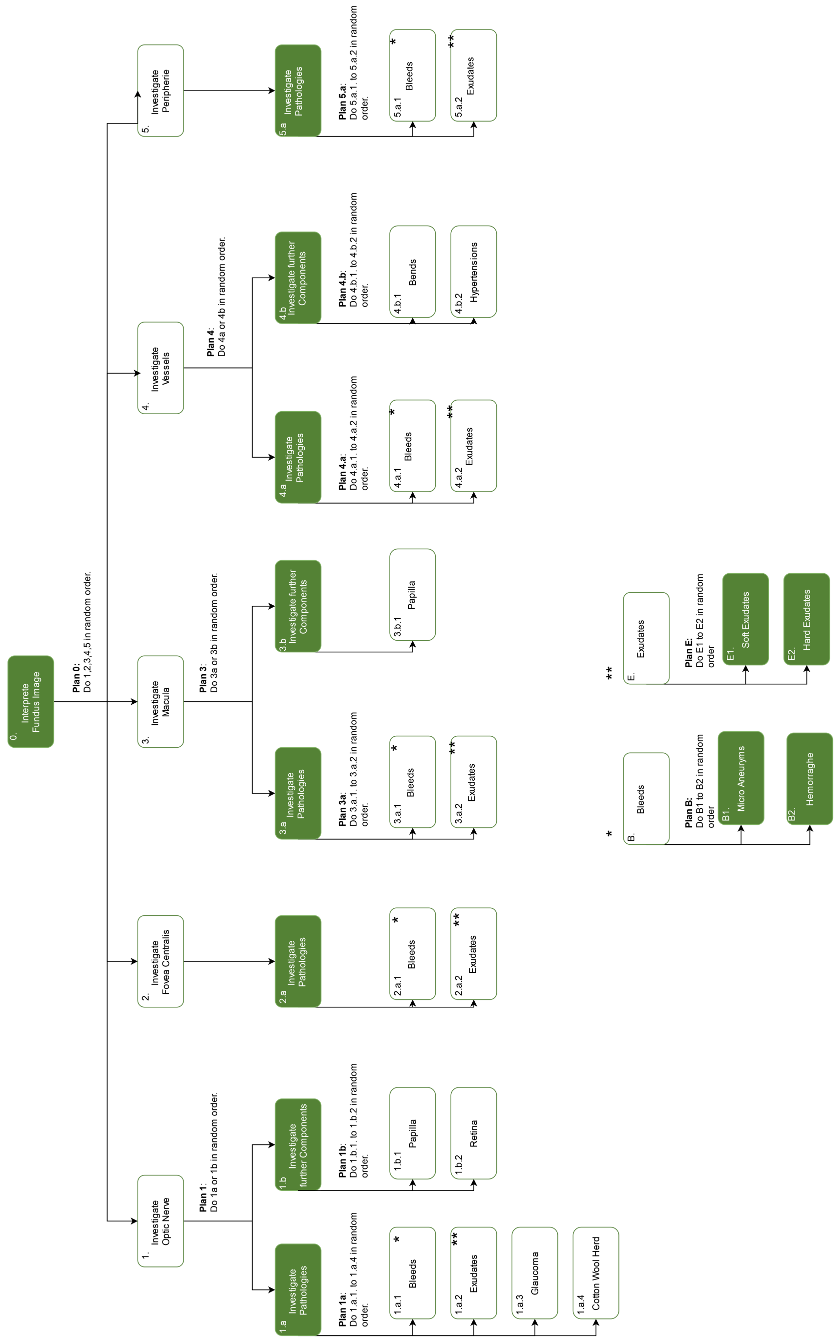

Figure S1: Overview of the hierarchical task analysis

2 EVALUATION STAGE - INTERVIEW GUIDELINE

The interview guidelines was applied in the evaluation stage to ensure a coherent briefing of participants and execution of interviews. The guideline consists of a briefing text for the interviewer to read to the participant and the interview questions. The questions are divided into four main parts: Warm-up and introduction, main questions, summary questions and an add-on question to as for anything the participant would like to add. The guidelines consists of 29 questions.

Evaluation Study – Interview Guideline

Inform the participant:

- To be able to evaluate the interview later, a recording will be made of the conversation and later, during a task, also of the screen. For this I would ask for your consent.
- The purpose of the interview is to get more information about your experience with the learning tool for ophthalmology.
- The interview is held in the context of a master thesis at {Anonymized Institutes}.
- The interview is voluntary and can be terminated at any time without negative consequences for you.
- The data will be analyzed anonymously, so that no conclusions can be drawn about your person.
- The anonymized results are used in this master thesis as well and other academic publications and reports.
- It is exclusively about your experiences, therefore there are no wrong answers.
- Feel free to ask if you have any questions or if something is unclear.

Participants read and sign the consent form.
The interview is recorded and as it progresses, notes are taken on statements that stand out.

**Warm-up**	1. How old are you?<br>2. In what year of training are you?
**Introduction**	3. Can you describe how interpreting Fundus images is taught to you in your training?<br>4. Can you describe how you receive feedback for your interpretation of fundus images during training?
**General**	3. Can you describe your experience with interpreting the task with the learning tool?<br>4. When you signed up for testing a learning tool to support the learning of interpreting fundus images, what did you expect?<br>a. Is the learning tool aligning with your expectations?<br>i. Yes/No: How so?<br>5. How did you experience to input pathologies such as micro aneurysms or hard exudates, into the learning tool?<br>6. Was the interpretation task clear for you? For example, how to input pathologies, or how to change between pathologies?<br>7. Would you say that interpreting fundus images would support you with learning how to interpret fundus images in your medical training?<br>8. How did you experience the feedback that the learning tool gave you after you input the pathologies?

Figure S2: Interview guideline of the evaluations study - Page 1

	9. Was the feedback given to you helpful in your learning process? For example:<br>a. The overview of feedback per pathology type<br>b. The split in correct, missed, and incorrect pathologies?<br><br>10. If you think about how the interpretation of fundus images is taught in your training now and in your studies: Would this learning tool be a helpful add-on to support you in learning how to interpret fundus images?<br><br>a. If Yes: How so? How is it supporting you, in a way that the current methods are not?<br>b. If No: How would it need to be so that it does support, you?
**Cognitive Load**	11. You were asked a question about the mental effort while using the learning tool. How did you experience answering this question?<br><br>12. How was your mental effort during using the learning tool?<br>a. If high: What contributed to your mental effort?<br><br>13. Did you feel like you must keep many things in mind simultaneously?<br><br>14. How complex was it to interpret the fundus image?<br><br>15. How did you experience finding important information in the learning tool?<br><br>16. How does the design of the learning tool effect your learning experience with the software?<br><br>17. Did you experience difficulties in linking information of the learning tool?
**Usability**	18. You were asked a set of questions about how easy the learning tool is to use and how well understandable it is. How did you experience answering this set of questions?<br><br>19. Did you experience difficulties in understanding your tasks when using the learning tool?<br><br>20. How complex is the learning tool for you? Why?<br><br>21. How was your initial understanding of using the learning tool? Did you need time to understand the design?<br><br>22. Did you find any inconsistencies in the learning tool? Where?
**Key Components for E-Learning**	23. How independent do you think you can use this learning tool to learn the interpretation of fundus images?<br>a. How does it compare to the independence in your current medical training?

Figure S3: Interview guideline of the evaluations study - Page 2

	24. How interactive do you find the learning tool?<br>a. How does it compare to the interactivity in your current medical training?<br><br>25. How well tailored was the feedback of the learning tool to you?<br>a. How does it compare to the feedback in your current medical training?<br><br>26. What is your opinion on the feature to upload your own examples in the learning tool?
**Summary**	27. In your opinion, how suitable is the learning tool to support you with learning how to interpret fundus images?<br><br>28. In general, how does this learning tool compare to how the interpretation of fundus images is taught in your current medical training?
**Add-on**	29. Would you like to add something?

Figure S4: Interview guideline of the evaluations study - Page 3
